# Nasolabial Soft Tissue Changes Following Maxillary Expansion: Influence of Baseline Morphology

**DOI:** 10.3390/dj14100640

**Published:** 2026-10-05

**Authors:** Karlina Kienkas, Gundega Jakobsone, Maksims Zolovs

**Affiliations:** 1 Department of General Dentistry, Institute of Stomatology, Riga Stradins University, LV-1007 Riga, Latvia; 2 Statistics Unit, Riga Stradins University, LV-1007 Riga, Latvia; 3 Institute of Life Sciences and Technology, Daugavpils University, LV-5401 Daugavpils, Latvia

**Keywords:** maxillary expansion, palatal rugae, facial soft tissues

## Abstract

**Objectives**: To determine whether intraoral and nasolabial changes after maxillary expansion (ME) can be predicted based on pretreatment morphology. **Methods**: The treatment group included 10–16-year-old adolescents who received treatment with RPE or MARPE. The RPE group comprised 13 females and nine males with a median age of 13 (interquartile range [IQR]: 11.1–13.8) years, and the MARPE group comprised 13 females and eight males with a median age of 14.4 (IQR: 13.2–15.2) years. Pre-treatment and post-expansion facial and intraoral scans were analysed. Linear regression was performed to assess whether pretreatment parameters were associated with differences in the change in parameters, adjusting for age, sex, and type of ME appliance. **Results**: The initially increased nostril widths were associated with a greater increase in the anatomical nose width, compared to the patients with decreased nostril widths, by 1.8 mm (*p* = 0.014) and 1.5 mm (*p* = 0.039) for the right and left nostrils, respectively. Individuals with reduced nose height (n-sn) experienced greater expansion in the medial rugae width (Tm3), by 0.9 mm (*p* = 0.001) and 1.3 mm (*p* = 0.002), compared to the medium- and increased-nose-height groups, respectively. Initially increased medial rugae width was associated with greater increase after ME. On the contrary, the increased maxillary base and intermolar widths were associated with less increase in those parameters. **Conclusions**: Initially larger distance between the third rugae was associated with a greater increase in the distance after ME, and a smaller distance in the maxillary width was associated with a greater increase after expansion. Larger initial nostril size was associated with a greater increase in the anatomical nose width after ME.

## 1. Introduction

Maxillary expansion (ME) has become a widely used treatment modality required in various clinical situations, ranging from a lack of space in the dental arch to improvement in upper airway permeability [1] and reduction in the severity of OSA [2].

Treatment effects are often assessed using CBCT [3,4,5]. However, given radiation concerns, intraoral scanning has emerged as a promising, non-invasive alternative. Kinzinger et al. suggested a close correlation between the opening of the maxillary suture in CBCT and changes in dental casts [6]. This finding was supported by previous studies [7,8,9], which highlighted changes in the palatal rugae following maxillary expansion.

Beyond dental and skeletal changes, research indicates that ME has an impact on facial aesthetics [10], and the mouth and nasolabial changes are clinically relevant and important for treatment planning [10,11]. The nose has a crucial role in overall facial beauty [12], and even small changes in nasal soft tissues could contribute to enhancements in respiratory function [13]. While studies show that patients with crossbite exhibit distinct facial parameters compared to those with normal occlusion [14], the predictability of post-expansion changes based on initial morphology remains poorly defined.

The choice of ME appliance often varies; it is influenced by subjective clinician preferences, as well as objective assessments of midpalatal suture maturation [15]. However, regardless of the device chosen, previous studies have often overlooked the natural growth occurring during the ME retention period. To objectively assess facial soft tissue changes following ME, age and sex must be considered as potential confounding factors, as there are distinct differences in facial growth between males and females [16,17,18,19], and age affects the parameters of the facial soft tissues [16].

The aim of the present study is to determine whether intraoral and nasolabial changes can be predicted based on pretreatment morphology, while accounting for appliance type and age- and sex-related growth differences.

## 2. Materials and Methods

### 2.1. Study Design

This prospective controlled cohort study aimed to assess whether initial intraoral and facial morphology were associated with changes in parameters, adjusting for age, sex, and type of ME appliance.

### 2.2. Participants

The study was conducted in accordance with the Declaration of Helsinki, and the study protocol was approved by the Ethics Committee of Riga Stradiņš University (RSU; NR.24/28 June 2018). Written informed consent was obtained from the participants’ parents/guardians. The treatment group included 10–16-year-old adolescents who visited RSU Institute of Stomatology, Department of Orthodontics between January 2020 and December 2021 and received treatment with RPE or MARPE, dictated by individual clinical considerations. The RPE group comprised 13 females and 9 males with a median age of 13 (interquartile range [IQR]: 11.1–13.8) years, and the MARPE group comprised 13 females and 8 males with a median age of 14.4 (IQR: 13.2–15.2) years.

### 2.3. Intervention

In the RPE group, a tooth-borne expander with custom-designed printed metal bands was anchored to the maxillary first permanent premolars and molars. In the MARPE group, the expander was attached to the bands on first permanent molars and two palatal miniscrews (8 mm × 1.7 mm, OrthoEasy^®^ Pal, Forestadent, Pforzheim, Germany), placed paramedian in the third palatal rugae region. Both groups had a 10 mm expansion screw (Forestadent, Pforzheim, Germany). The appliances were activated with a quarter turn (0.2 mm) per day until the palatal cusps of the maxillary first molars touched the buccal cusps of the mandibular first molars. Once the required expansion was achieved, the appliance was left in the mouth over a retention period of at least 5 months.

**Figure 1:**
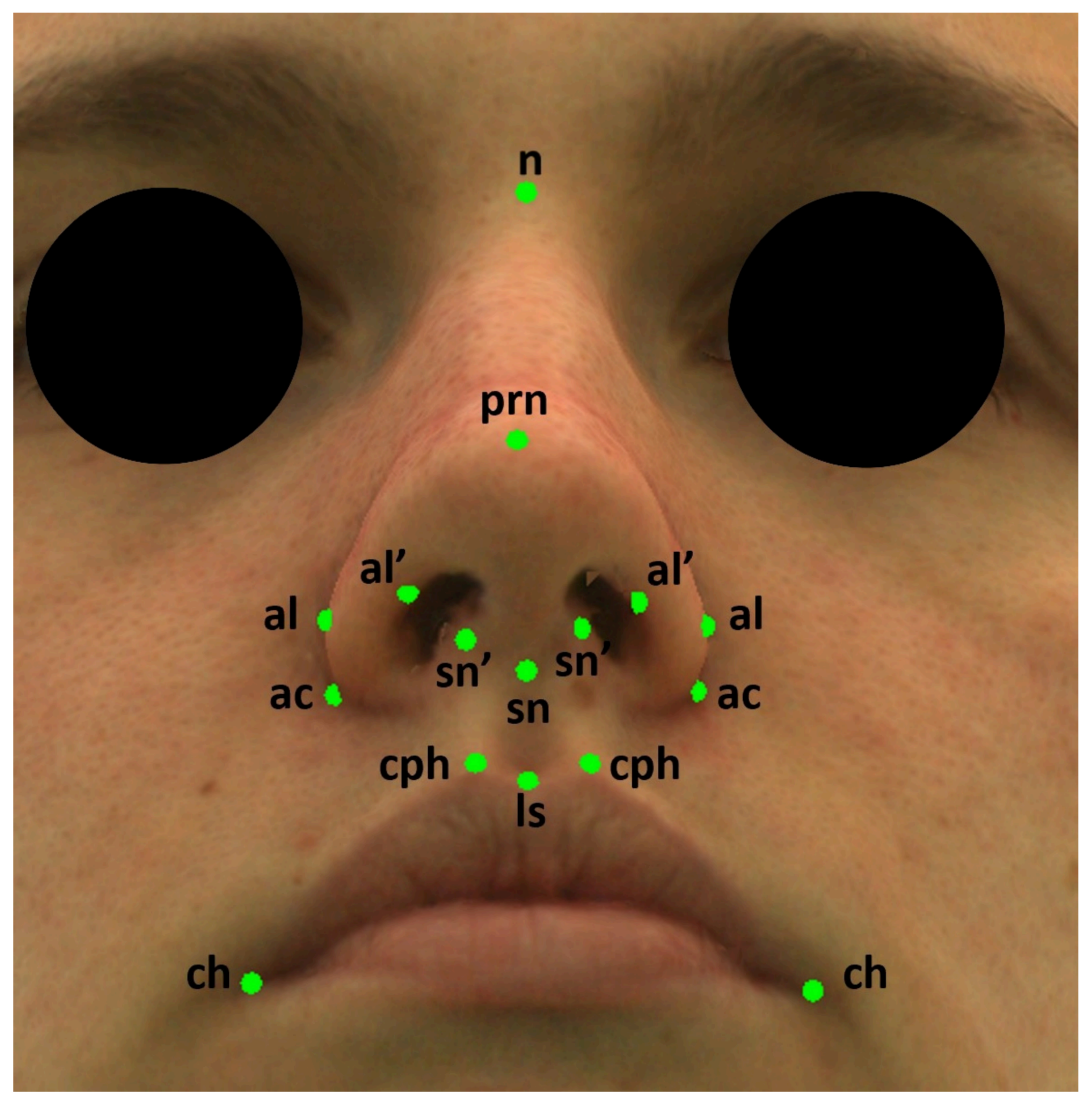
The facial landmarks used in the analysis.

Intraoral and facial scans were obtained by two experienced operators at two timepoints: pre-treatment (T0) and an average 6 months after the start of maxillary expansion (T1). The median period between T0 and T1 was 6.34 months (IQR 5.79–6.99). Facial surface scans were acquired using a 3dMDface imaging system (3dMD, Atlanta, GA, USA), which uses multiple cameras to capture a person’s face from ear to ear in 1.5 milliseconds. Scans were taken with a neutral facial expression and head in natural head position with the same light settings and ambient conditions. The scans were landmarked using 3dMD Vultus software (version 2.5.0.1); landmarks were positioned as described by Farkas [20] (Table 1, Figure 1). The landmarks were assessed using the x-, y-, and z-coordinates, and the distances and angles (Table 2) between the coordinates of the landmarks were measured.

**Figure 2:**
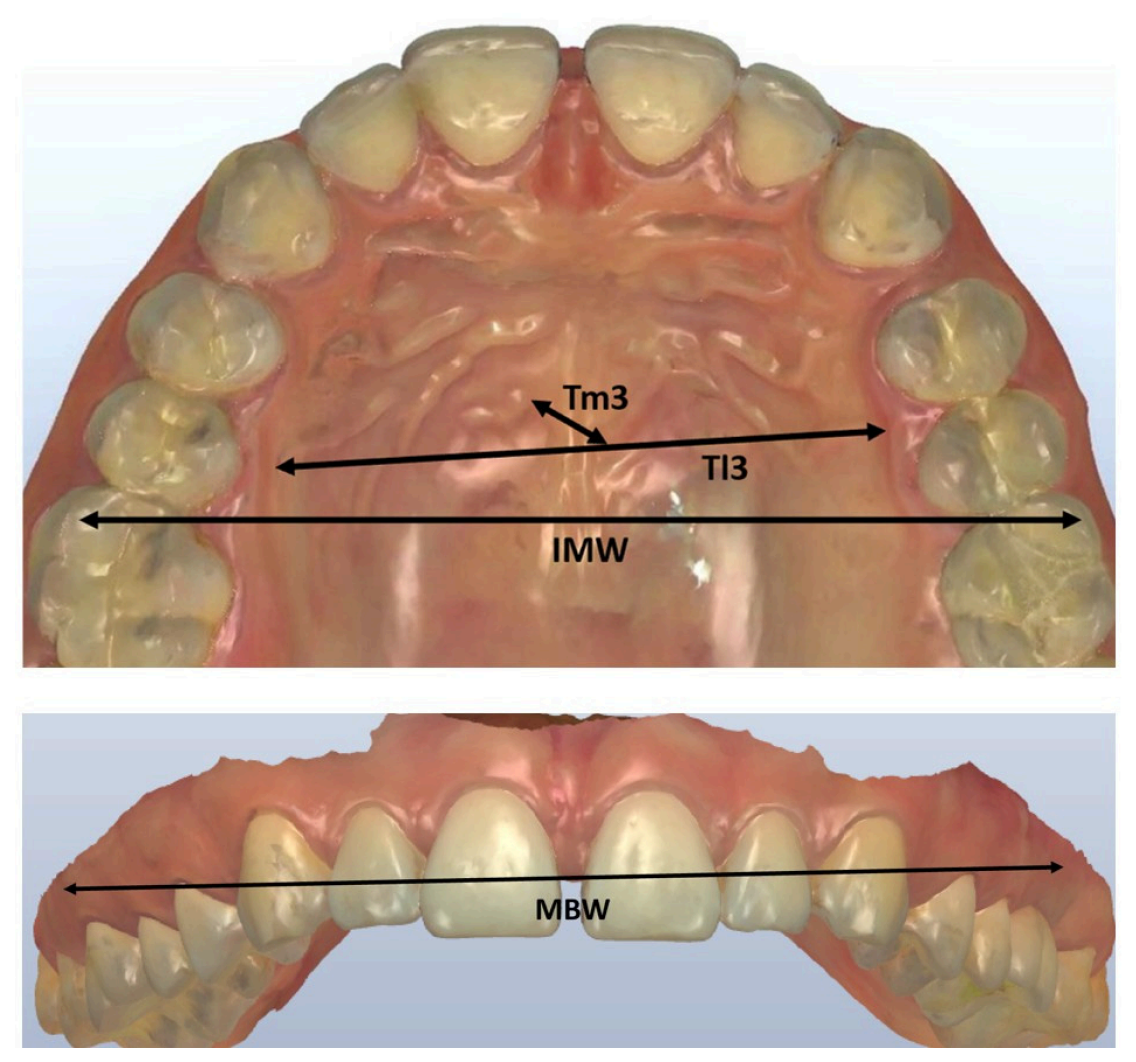
Intraoral measurements.

Intraoral scans were acquired with an intraoral scanner (TRIOS, 3Shape, Copenhagen, Denmark) and saved as STL files. Scans were analysed in the 3Shape Ortho Analyzer software (version 1.9.3.4.), and landmark-based analysis was performed using four linear measurements (Table 2, Figure 2). Medial (Tm3) and lateral (Tl3) rugae widths, intermolar width (IMW) and maxillary base width (MBW) were measured. MBW was measured as the distance between two points on the left and right sides, where a line drawn perpendicular to the occlusal plane, at the projection of the centre of the maxillary first molar clinical crown on vestibular surface, touches maximum bone contour [21]. All landmarking and measurements were performed by a single operator.

### 2.4. Method Error

The intra-operator reliability for intraoral measurements was conducted with 20 scans that were remeasured 2 weeks apart. Intraclass correlations (two-way, single measurements, absolute agreement) were calculated; coefficients ranged from 0.91 to 0.99.

The intra-operator reliability of the facial scans was assessed by a single operator landmarking 20 scans with 16 landmarks at 2-week intervals. The errors in landmark coordinate identification were categorised as follows [22]: <0.5 mm, high reproducibility; <1 mm, moderate reproducibility; and >1 mm, poor reproducibility. Of the 48 coordinates, 43 were highly reliable and 5 were moderately reliable. Furthermore, 3 out of 5 moderately reliable coordinates were on the Y-axis. The mean facial landmark coordinate identification error was 0.3 ± 0.32 mm.

### 2.5. Statistical Analysis

Each pretreatment parameter was stratified into three categories based on the sample mean (M) and standard deviation (SD): reduced (<M–SD), medium (M–SD to M + SD), and increased (>M + SD).

Linear regression analysis was performed to assess whether stratified pretreatment parameters were associated with differences in the change (Δ) in parameters, adjusting for age, sex, and type of ME appliance. For each facial parameter, a separate linear regression model was fitted. Model assumptions, including linearity, homoscedasticity, normality of residuals, and absence of multicollinearity were assessed.

No formal a priori sample size calculation was performed. The sample size was determined by the number of eligible participants recruited during the predefined study period. To characterise the statistical sensitivity of the regression analyses, a sensitivity analysis was conducted for the overall multiple regression model. Assuming five non-intercept terms, a two-sided significance level of 0.05, and 80% power, the achieved sample size (n = 43) provided sensitivity to detect Cohen’s values of approximately 0.346.

## 3. Results

Table 3 shows facial and intraoral parameters in MARPE and RPE groups before ME. The initial variables are presented as marginal means, calculated by linear regression, adjusting for age and sex. As the initial extraoral and intraoral parameters did not differ between MARPE and RPE, the data were pooled together for further linear regression analysis.

The marginal means of facial variables after ME are summarised in Table 4. After ME, both groups presented with similar intraoral and extraoral parameters. The only statistically significant difference between groups was recorded for the maxillary base width, with MARPE showing 2.2 mm greater width than the RPE group (*p* = 0.019).

Several baseline parameters demonstrated significant associations with facial and intraoral changes following ME; all statistically significant associations are presented in Table 5.

The initially increased nostril widths were associated with a significantly greater increase in the anatomical nose width, compared to the patients with decreased nostril widths, by 1.8 mm (CI: 0.39–3.19; *p* = 0.014) and 1.5 mm (CI: 0.08–2.94; *p* = 0.039) for right and left nostrils, respectively.

For patients with initially increased left nostril width, morphological nose width increased more than for patients with medium (by 0.7 mm (CI: 0.19–1.25; *p* = 0.009)) and reduced nostril width (by 1.1 mm (CI: 0.47–1.81; *p* = 0.002).

In both reduced- and increased-philtrum-width groups, the changes produced by ME were smaller compared to the medium-size-philtrum-width group, by 0.9 mm (CI: −0.08–1.91; *p* = 0.071) and 1.3 mm (CI: 0.24–2.38; *p* = 0.018), respectively.

Individuals with reduced nose height experienced greater expansion in the medial rugae width (Tm3), by 0.9 mm (CI: 0.39–1.39; *p* = 0.001) and 1.3 mm (CI: 0.52–2.13; *p* = 0.002), compared to medium- and increased-nose-height groups, respectively.

Initially increased medial rugae width was associated with greater increase as a result of ME. On the contrary, the initially increased maxillary base and intermolar widths were associated with less increase in those parameters.

## 4. Discussion

In this study, we aimed to assess the influence of the initial patient morphology on the treatment effects of maxillary expansion (ME) while accounting for age, sex and appliance type. Our results confirmed the assumption that the initial parameters could have an effect on the changes produced by ME. Although linear regression was intended to explore associations between baseline facial morphology and subsequent changes, multiple comparisons may increase the risk of chance findings. The observed associations should therefore be interpreted as exploratory and warrant confirmation in future studies with prespecified hypotheses.

Initially wider nostrils were associated with a greater increase in anatomical nose width, suggesting changes in nasal appearance. It must be noted that nostrils are of central importance in the appearance of the nose, and even minimal changes in the nasal base influence nasal shape [23]. Therefore, patients with initially wide nostrils should be carefully examined before ME and warned about potential nasal changes.

Right and left nostril widths showed different associations with facial parameters changes after ME. Differences in left- and right-side changes may be due to the great majority of people presenting with some degree of facial asymmetry [24]. Narrower right nostril width before treatment was associated with a greater increase in right nostril width. While males with crossbite have been reported to present with significantly narrower nostrils than individuals with normal transversal occlusion [14], whether an increase in external nostril dimensions translates to clinically significant improvement remains to be evaluated. Potentially, an increase in nostril size, especially for those with narrow nostrils, may provide functional benefits, as an increase in the minimum cross-sectional area of the anterior part of the nose, surrounded mainly by soft tissues (nostrils), can improve the subjective sensation of nasal function [25] and reduce nasal respiratory resistance [13]. However, in most individuals, nasal resistance is not determined by a single constricted segment; rather, it represents the cumulative resistance across all segments of the nasal airway [26]. Therefore, any potential functional implications remain theoretical hypotheses that require future investigation.

Patients with increased medial rugae width experienced a greater increase in the rugae width. This finding may indicate less changes in the shape of the palate for patients with an initially narrow palate and question the overall palatal volume changes after ME for patients with a high, narrow palate, as palatal height tends to decrease during ME [27]. This may also suggest that patients with a narrow palate experience less increase in nasal cavity, as for every millimetre of midpalatal suture opening, the nasal cavity width increases by 0.57 mm [28]. Additionally, a greater increase in the medial rugae width was observed for the patients with decreased middle face height, expressed by reduced nose height (n-sn). This finding confirms the previously mentioned pattern of greater medial rugae width increase for individuals with increased rugae width, as hypo-divergent facial patterns are reported to have greater medial rugae width [29]. A smaller increase in medial rugae width for patients with already decreased medial rugae width may suggest limited benefits from ME for patients with high, narrow arched palate in adolescence. As palatal shape adapts more at a younger age [6], earlier ME would be advisable for those with high, narrow palates.

Patients with reduced mouth width experienced a greater increase in morphological nose width (*p* = 0.037) and left nostril width (*p* = 0.046), suggesting beneficial facial changes for patients with crossbite, as they tend to have reduced left nostril and mouth widths prior to treatment [14].

Overall, the inconsistent association patterns observed between initial morphology and changes during ME may be ascribed to the well-established influence of both genetic and environmental factors on facial morphology [30,31,32]. The nasal region shows high heritability in European populations [31]. Djordjevic et al. suggested that genetic factors can explain more than 70% of the phenotypic facial variations in the width, height and prominence of the nose [33]. Although several association patterns were statistically significant, some differences fall below reported measurement errors while others clearly exceed the margin of error. Nevertheless, clinical significance should be interpreted with caution and only as a potential predictive pattern.

No differences in facial parameters were found between the MARPE and RPE groups after ME. MARPE produced greater intraoral changes, though only the difference in maxillary width reached statistical significance. The result of the present study suggests that the changes in the maxillary dimensions are not directly linked with the facial changes, as reported in previous studies [34,35]. Soft tissues only partially express skeletal movements, as patients with different skeletal expansion amounts exhibit similar soft tissue changes [36]. It could be assumed that the response of soft tissues of the alar region could follow skeletal expansion up to a certain threshold [37], which may also explain why different types of ME appliances yield similar facial soft tissue changes.

### Limitations

The main limitation of the current study is the short-term observation period. Previous long-term research has demonstrated that there are no statistically significant changes in facial soft tissues after ME compared to controls [38]. Furthermore, the complete elimination of facial expressions during examination could not be guaranteed. Maal et al. reported a mean variation of 0.25 mm between images employing stereophotogrammetry [39]. While age was included as a covariate in all regression models, individual variation in growth velocity during the observation period was not tracked via skeletal maturation staging.

No formal a priori sample size calculation was performed, and the achieved sample size was determined by participant availability during the study period. Consequently, small and moderate associations may have remained undetected, whereas estimates derived from sparsely populated baseline categories may be imprecise. The regression findings should therefore be interpreted as exploratory and require confirmation in larger, prospectively powered studies.

## 5. Conclusions

- Initially larger distance between the third palatal rugae was associated with a greater increase in the distance after ME, and a smaller distance in the maxillary width was associated with a greater increase after expansion.
- Baseline facial parameters were not associated with post-expansion changes in the same structures, except for the philtrum and right nostril width.
- Larger initial nostril size was associated with a greater increase in anatomical nose width after expansion.

## Figures and Tables

**Table 1:** Facial landmark definitions.

Landmark	Definition
n (nasion)	The point in the midline of both the nasal root and nasofrontal suture
al (alare) ^R,L^	The most lateral point on the alar contour
ac (alar curvature) ^R,L^	The most lateral point in the curved baseline of the ala
sn (subnasale)	The midpoint of the angle at the columella base where the lower border of the nasal septum and surface of the upper lip meet
prn (pronasale)	The most protruded point of the apex nasi
al’ (alare’) ^R,L^	Marking level at the midportion of the alae
sn’ (subnasale’) ^R,L^	The midpoint of the columella crest
cph (crista philtri) ^R,L^	The point on the elevated margin of the philtrum just above the vermilion line
ch (cheilion) ^R,L^	The point at the labial commissure
ls (labiale superius)	The midpoint of the upper vermilion line

^R^ right; ^L^ left.

**Table 2:** Definitions of the measured parameters.

**Linear Facial Measurements (mm)**	
al-al	Morphological nose width (alar width)
ac-ac	Anatomical nose width
n-sn	Nose height
n-prn	Nasal dorsum length/nose length
al’-sn’ ^R,L^	Nostril width
cph-cph	Philtrum width
ch-ch	Mouth width
sn-ls	Height of the cutaneous upper lip
**Intraoral measurements (mm)**	
Tm3	Distance between medial points of third palatal rugae
Tl3	Distance between lateral points of third palatal rugae
IMW	Distances between mesiobuccal cusp tips of upper first permanent molars
MBW	Maxillary base width

^R^ right; ^L^ left.

**Table 3:** Nasolabial and intraoral parameters before treatment.

Parameter	MARPE	RPE	*p* Value
	Marginal mean (95% CI), mm		
	**Linear nasolabial measurements**		
**al-al**	32.8 (31.7–33.9)	33.4 (32.4–34.5)	0.421
**ac-ac**	31.7 (30.5–32.8)	31.9 (30.7–33)	0.824
**al’-sn’ ^R^**	6.9 (6.5–7.3)	7.1 (6.7–7.5)	0.526
**al’-sn’ ^L^**	7 (6.5–7.4)	7.2 (6.7–7.6)	0.581
**n-prn**	41.4 (39.9–42.8)	40.7 (39.3–42.1)	0.515
**n-sn**	48.7 (47.3–50.1)	48.1 (46.8–49.4)	0.558
**cph-cph**	12.4 (11.7–13.2)	11.7 (11–12.5)	0.209
**ch-ch**	44.7 (43.4–46)	44.2 (42.9–45.5)	0.593
**sn-ls**	14.6 (13.5–15.7)	13.4 (12.3–14.5)	0.146
**Intraoral measurements**			
**Tm3**	2.4 (1.8–2.9)	2.3 (1.9–2.8)	0.939
**Tl3**	22.6 (21.4–23.7)	22.7 (21.6–23.7)	0.865
**IMW**	49.1 (47.6–50.7)	48.6 (47.2–50)	0.624
**MBW**	56.3 (54.9–57.7)	55.3 (54.1–56.6)	0.319

Data are shown as marginal mean (adjusted mean after age and sex control) and 95% confidence interval (CI). Linear and intraoral measurements are in millimetres. ^R^ right; ^L^ left.

**Table 4:** Nasolabial and intraoral parameters after treatment.

Parameter	MARPE	RPE	*p* Value
	Marginal mean (95% CI), mm		
	**Linear nasolabial measurements**		
**al-al**	34.2 (33.2–35.2)	34.1 (33.1–35.1)	0.916
**ac-ac**	32.7 (31.6–33.8)	31.7 (30.6–32.7)	0.214
**al’-sn’ ^R^**	7.5 (7.1–7.9)	7.7 (7.3–8.1)	0.442
**al’-sn’ ^L^**	7.7 (7.2–8.2)	7.8 (7.3–8.3)	0.826
**n-prn**	41.8 (40.3–43.3)	41.2 (39.8–42.7)	0.586
**n-sn**	49.6 (48.1–51)	49 (47.6–50.4)	0.607
**cph-cph**	12.1 (11.2–13.1)	11.9 (11.1–12.8)	0.772
**ch-ch**	47.1 (45.5–48.6)	46.9 (45.4–48.3)	0.853
**sn-ls**	14.2 (13.2–15.3)	13.4 (12.4–14.4)	0.253
**Intraoral measurements**			
**Tm3**	4.1 (3.3–4.8)	3.2 (2.5–3.8)	0.083
**Tl3**	27.6 (26.4–28.9)	27.1 (26–28.3)	0.545
**IMW**	56.7 (55.3–58)	54.9 (53.6–56.1)	0.063
**MBW**	62.9 (61.6–64.2)	60.7 (59.6–61.9)	**0.019**

Data are shown as marginal mean (adjusted mean after age and sex control) and 95% confidence interval (CI). Linear and intraoral measurements are in millimetres. ^R^ right; ^L^ left.

**Table 5:** Differences in changes according to categories of baseline morphology.

Outcome	Parameter		B (mm)	β	95% CI of B	t	*p* Value	Adj. R^2^	Model *p*
**Δ al-al**	**al’-sn’ ^L^ T0**	2–1	0.4	0.559	−0.12–0.96	1.570	0.125	0.327	0.001
		3–1	1.1	1.522	0.47–1.81	3.427	**0.002**		
		3–2	0.7	0.963	0.19–1.25	2.790	**0.009**		
	**ch-ch T0**	2–1	−0.6	−0.798	−1.16–−0.04	−2.165	**0.037**	0.213	0.015
		3–1	−0.3	−0.380	−0.99–0.42	−0.814	0.421		
		3–2	0.3	0.418	−0.24–0.86	1.150	0.258		
**Δ ac-ac**	**al’-sn’ ^R^ T0**	2–1	0.8	0.527	−0.27–1.89	1.520	0.137	0.299	0.002
		3–1	1.8	1.163	0.39–3.19	2.590	**0.014**		
		3–2	1.0	0.635	−0.15–2.1	1.760	0.087		
	**al’-sn’ ^L^ T0**	2–1	0.3	0.212	−0.82–1.47	0.575	0.569	0.282	0.004
		3–1	1.5	0.980	0.08–2.94	2.136	**0.039**		
		3–2	1.2	0.768	0.07–2.3	2.150	**0.039**		
	**sn-ls T0**	2–1	0.0	0.024	−1.23–1.29	0.058	0.954	0.316	0.002
		3–1	−1.5	−0.961	−2.96–0.01	−2.016	0.051		
		3–2	−1.5	−0.985	−2.64–−0.39	−2.730	**0.010**		
**Δ al’-sn’ ^R^**	**al’-sn’ ^R^ T0**	2–1	−0.7	−1.048	−1.25–−0.25	−3.031	**0.004**	0.302	0.002
		3–1	−0.9	−1.318	−1.59–−0.29	−2.938	**0.006**		
		3–2	−0.19	−0.269	−0.71–0.33	−0.75	0.459		
**Δ al’-sn’ ^L^**	**ch-ch T0**	2–1	−0.5	−0.766	−0.91–−0.01	−2.065	**0.046**	0.202	0.019
		3–1	−0.4	−0.665	−0.97–0.17	−1.415	0.165		
		3–2	0.1	0.101	−0.38–0.51	0.276	0.784		
**Δ cph-cph**	**cph-cph T0**	2–1	0.9	0.728	−0.08–1.91	1.859	0.071	0.122	0.079
		3–1	−0.4	−0.315	−1.73–0.94	−0.600	0.552		
		3–2	−1.3	−1.043	−2.38–−0.24	−2.468	**0.018**		
**Δ ch-ch**	**ac-ac T0**	2–1	−0.1	−0.047	−2.20–1.99	−0.104	0.918	0.199	0.020
		3–1	2.1	0.927	−0.55–4.77	1.607	0.117		
		3–2	2.2	0.975	0.39–4.04	4.040	**0.019**		
	**cph-cph T0**	2–1	2.2	0.983	0.54–3.92	2.677	**0.011**	0.229	0.011
		3–1	1.2	0.506	−1.12–3.42	1.029	0.310		
		3–2	−1.1	−0.477	−2.91–0.74	−1.200	0.236		
**Δ Tm3**	**n-sn T0**	2–1	−0.9	−1.241	−1.39–−0.39	−3.603	**0.001**	0.502	<0.001
		3–1	−1.3	−1.848	−2.13–−0.52	−3.346	**0.002**		
		3–2	−0.4	−0.607	−1.07–0.21	−1.38	0.176		
	**n-prn T0**	2–1	−0.7	−0.937	−1.24–0.1	−2.393	**0.023**	0.436	<0.001
		3–1	−1.2	−1.640	−1.94–0.4	−3.092	**0.004**		
		3–2	−0.5	−0.703	−1.01–0.05	−2.020	0.052		
	**Tm3 T0**	2–1	0.0	0.012	−0.57–0.59	0.030	0.976	0.422	<0.001
		3–1	1.2	1.682	0.21–2.19	2.476	**0.019**		
		3–2	1.2	1.670	0.36–2.03	2.920	**0.006**		
**Δ Tl3**	**IMW T0**	2–1	−1.0	−0.668	−2.51–0.44	−1.426	0.163	0.035	0.294
		3–1	−2.0	−1.308	−4.03–−0.03	−2.059	**0.047**		
		3–2	−1.0	−0.640	−2.45–0.46	−1.39	0.175		
**Δ IMW**	**IMW T0**	2–1	−1.9	−1.034	−3.5–−0.41	−2.565	**0.015**	0.286	0.005
		3–1	−3.3	−1.721	−5.35–−1.16	−3.150	**0.003**		
		3–2	−1.3	−0.688	−2.83–0.23	−1.730	0.093		
	**MBW T0**	2–1	−1.1	−0.559	−2.58–0.46	−1.415	0.166	0.195	0.028
		3–1	−2.4	−1.276	−4.56–−0.27	−2.284	**0.029**		
		3–2	−1.4	−0.717	−2.98–0.27	−1.700	0.099		
**Δ MBW**	**IMW T0**	2–1	−0.9	−0.583	−2.1–0.35	−1.447	0.157	0.287	0.005
		3–1	−2.5	−1.693	−4.18–−0.87	−3.100	**0.004**		
		3–2	−1.7	−1.110	−2.86–−0.45	−2.790	**0.009**		
	**MBW T0**	2–1	−0.1	−0.046	−1.23–1.09	−0.120	0.905	0.243	0.012
		3–1	−1.8	−1.192	−3.42–−0.14	−2.201	**0.035**		
		3–2	−1.7	−1.146	−2.95–−0.47	−2.800	**0.008**		

Δ—delta (difference between T1 and T0). Each pretreatment parameter was stratified into three categories based on the sample mean (M) and standard deviation (SD): 1—reduced (<M−SD), 2—medium (M−SD to M + SD), and 3—increased (>M + SD). B—estimate (difference between reduced (1), medium (2), increased (3) groups in millimetres). β—the outcome-standardised contrast coefficient. ^R^ right; ^L^ left.

## Data Availability

The data presented in this study are available on request from the corresponding author.
